# Design and Modeling of a MEMS Dual-Backplate Capacitive Microphone with Spring-Supported Diaphragm for Mobile Device Applications

**DOI:** 10.3390/s18103545

**Published:** 2018-10-19

**Authors:** Néstor N. Peña-García, Luz A. Aguilera-Cortés, Max A. González-Palacios, Jean-Pierre Raskin, Agustín L. Herrera-May

**Affiliations:** 1 Departamento de Ingeniería Mecánica, DICIS, Universidad de Guanajuato, Carr. Salamanca-Valle de Santiago km 3.5 + 1.8 km, Palo Blanco, Salamanca, Guanajuato 36885, Mexico; aguilera@ugto.mx (L.A.A.-C.); maxg@ugto.mx (M.A.G.-P.); 2 Institute of Information and Communication Technologies, Electronics and Applied Mathematics (ICTEAM), Université catholique de Louvain (UCL), 1348 Louvain-la-Neuve, Belgium; jean-pierre.raskin@uclouvain.be; 3 Micro and Nanotechnology Research Center, Universidad Veracruzana, Calzada Ruiz Cortines 455, Boca del Río, Veracruz 94294, Mexico; leherrera@uv.mx; 4 Maestría en Ingeniería Aplicada, Facultad de Ingeniería de la Construcción y el Hábitat, Universidad Veracruzana, Calzada Ruíz Cortines 455, Boca del Río, Veracruz 94294, Mexico

**Keywords:** capacitive microphone, dual backplate, FEM model, electret condenser microphones, spring-supported diaphragm, Sandia Ultra-Planar Multi-level MEMS Technology V (SUMMiT V) fabrication process

## Abstract

New mobile devices need microphones with a small size, low noise level, reduced cost and high stability respect to variations of temperature and humidity. These characteristics can be obtained using Microelectromechanical Systems (MEMS) microphones, which are substituting for conventional electret condenser microphones (ECM). We present the design and modeling of a capacitive dual-backplate MEMS microphone with a novel circular diaphragm (600 µm diameter and 2.25 µm thickness) supported by fifteen polysilicon springs (2.25 µm thickness). These springs increase the effective area (86.85% of the total area), the linearity and sensitivity of the diaphragm. This design is based on the SUMMiT V fabrication process from Sandia National Laboratories. A lumped element model is obtained to predict the electrical and mechanical behavior of the microphone as a function of the diaphragm dimensions. In addition, models of the finite element method (FEM) are implemented to estimate the resonance frequencies, deflections, and stresses of the diaphragm. The results of the analytical models agree well with those of the FEM models. Applying a bias voltage of 3 V, the designed microphone has a bandwidth from 31 Hz to 27 kHz with 3 dB sensitivity variation, a sensitivity of 34.4 mV/Pa, a pull-in voltage of 6.17 V and a signal to noise ratio of 62 dBA. The results of the proposed microphone performance are suitable for mobile device applications.

## 1. Introduction

Microelectromechanical systems (MEMS) technology have allowed the development of microphones with characteristics such as a small size, low power consumption, reduced cost, high signal quality and good stability respect to variations of temperature and humidity [1,2]. These microphones can provide a substitute for conventional electret condenser microphones (ECM) in mobile electronics devices, including smartphones, laptops and tablets [3]. MEMS microphones are devices composed mainly of diaphragms that transform the sound pressure (i.e., acoustic wave) into electrical signals. In most applications, this pressure has small values from 200 µPa (20 dB) to 10 Pa (114 dB), which is measured in environments with high dc background atmospheric pressure close to 100 kPa. Generally, the audio bandwidth of interest has a frequency range from 20 Hz to 20 kHz [4]. The pressure of the acoustic wave interacts with the microphone diaphragm, causing deflections of the diaphragm. These deflections can be measured using different transduction principles: Piezoelectric, piezoresistive, optical and capacitive. MEMS microphones with capacitive sensing can use one or two rigid backplates and a diaphragm, which oscillates due to the incident pressure. Commonly, each microphone backplate has holes into which the air flows, generating diaphragm deflections. Both backplate and diaphragm form a capacitor that measures the diaphragm deflections through the capacitance shifts. 

Rombach et al. [5] fabricated the first differential MEMS microphone using two backplates and a square membrane to increase its sensitivity. This membrane (2000 µm × 2000 × µm 0.5 µm) registered a stress of 45 MPa, a sensitivity of 13 mV/Pa under a bias voltage of 1.5 V, an equivalent noise level of 22.5 dBA for an integration range from 20 Hz to 20 kHz. Iguchi et al. [6] developed a MEMS microphone using a silicon diaphragm (2100 µm × 2100 µm × 10 µm). This microphone had a frequency range from 30 Hz to 20 kHz, a sensitivity of −52 dBV/Pa considering a bias voltage of 39 V, a pull-in voltage of 170 V and an equivalent noise level of 47 dBA, and it supports a sound pressure of 20 Pa. Martin et al. [7,8] developed an analytical model of a MEMS dual-backplate microphone with a polysilicon diaphragm (460 µm diameter) for aeroacoustic measurements. This microphone is fabricated using the Sandia Ultra-Planar Multi-level MEMS Technology V (SUMMiT-V) process. The microphone has a resonant frequency of 178 kHz, a bias voltage of 9.3 V, a sensitivity of 0.39 mV/Pa and a noise floor of 41 dB/√Hz. [9] designed a MEMS capacitive microphone formed by a perforated aluminum diaphragm (500 µm × 500 µm × 3 µm). This microphone has a bandwidth up to 20 kHz and a sensitivity of 0.2 mV/Pa with a bias voltage of 105 V. Grixti et al. [10] created a mathematical model of MEMS microphone composed by a clamped square diaphragm (675 µm × 675 µm), which is based on the PolyMUMPs process. This microphone is supplied by a voltage of 6 V, achieving a sensitivity of 8.4 mV/Pa and a cut-off frequency of 10.5 kHz. Gharaei and Koohsorkhi [11] proposed an analytical model to design a MEMS microphone with a fungous-coupled diaphragm structure (460 µm diameter) that increased the diaphragm effective area, obtaining a parallel plates capacitor. This microphone has a pull-in voltage of 13 V, a sensitivity of 1.3 mV/Pa using a bias voltage of 11 V and a maximum frequency of 100 kHz. In addition, this microphone supports a sound pressure of 160 dB, which is suitable to be employed in aeroacoustic measurements. Zargarpour and Zarifi [12] presented a piezoelectric MEMS microphone for implantable hearing aid applications, considering a circular silicon diaphragm (350 µm diameter and 10 µm thickness). This microphone has a frequency range between 20 Hz and 20 kHz and a first resonant frequency of 332.87 kHz. Next, Zargarpour, Abdi and Bahador [13] designed the voltage amplifier circuit of the piezoelectric MEMS microphone using the 180 nm standard CMOS technology. With this circuit were achieved an amplification gain of 84.78 dB, an average power consumption of 0.216 mW and a noise level of 4.192 µVrms. However, several of these MEMS capacitive microphones use diaphragms whose deflections cause a high reduction of capacitor effective area and mechanical sensitivity. In order to increase the sensitivity of the MEMS dual-backplate capacitive microphones, we designed a circular polysilicon diaphragm (600 µm diameter and 2.25 µm thickness) supported by a novel array of fifteen polysilicon springs. This design increases the effective area of the diaphragm when it oscillates due to the sound pressure, which increases the capacitance shift between the diaphragm and dual-backplates. In addition, analytical and finite element method (FEM) models are developed to predict the electromechanical behavior of the proposed microphone.

## 2. Modeling and Design

In this section, the design of the microphone components and its operation principle are described along with the theoretical performance. In addition, analytical models to estimate the stiffness, deflection and first resonant frequency of the circular diaphragm are developed. Finally, the capacitance shift, output voltage and noise of the MEMS microphone are obtained.

### 2.1. Microphone Structure

**Figure 1:**
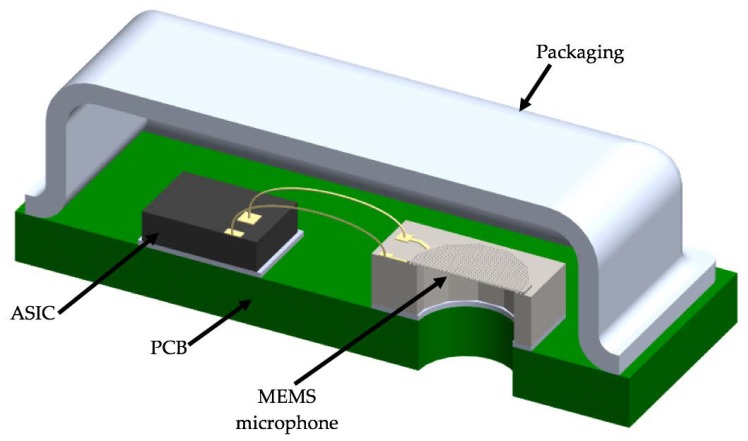
Schematic view of a MEMS microphone with the sound port in the substrate.

The MEMS microphone is formed by a circular polysilicon diaphragm (600 µm diameter and 2.25 µm thickness) that is located between two polysilicon plates with a hexagonal array of holes (see Figure 1). This microphone design is based on SUMMiT V surface micromachining process from Sandia National Laboratories [14]. The substrate below the polysilicon diaphragm of the microphone must be etched to allow the application of the sound pressure to the diaphragm. This pressure will cause diaphragm deflections that will be converted into electrical signals (i.e., capacitance variations between diaphragm and dual-backplates). The diaphragm and dual-backplates are the capacitor electrodes and using a bias voltage, the capacitance shifts can be measured with an application specific integrated circuit (ASIC). 

**Figure 2:**
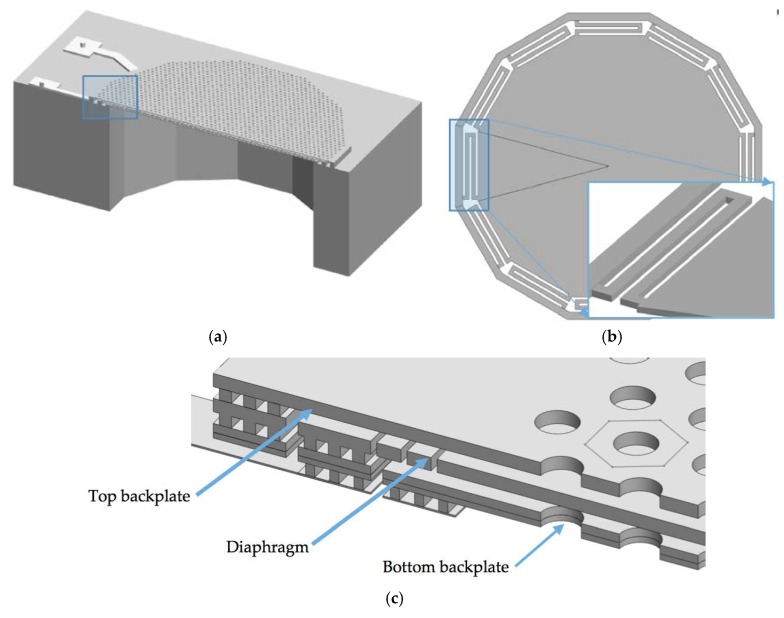
3D view of the MEMS microphone formed by (**a**) circular diaphragm and two backplates with holes and (**b**) springs array located on the diaphragm edge; (**c**) Detail of the cross-section view of the differential capacitive MEMS microphone.

The array of *n* polysilicon springs along the diaphragm edge enables a uniform movement of the diaphragm, increasing the diaphragm effective area with respect to a clamped diaphragm (see Figure 2). In addition, the space between springs creates some slits, which work as an air channel to allow the air flow between the back cavity and environment that avoids the diaphragm motion due the fluctuations of the atmospheric pressure. The springs will increase the capacitance shift and sensitivity of the microphone. Thus, these parameters can be controlled varying the compliance or stiffness of the diaphragm. 

**Figure 3:**
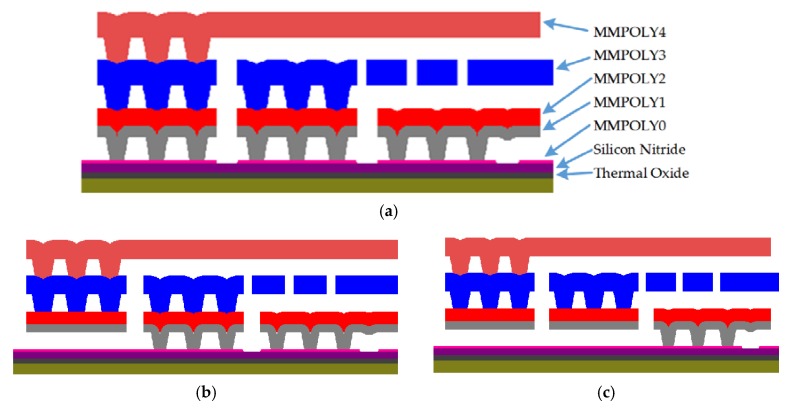
Schematic view of the microphone design based on the SUMMiT V surface-micromachining process. (**a**) Anchor structure of the diaphragm and backplates, and electrical connection of the (**b**) diaphragm and (**c**) bottom backplate of the microphone.

Figure 3 depicts the structural design of the MEMS microphone that considers three polysilicon layers. The first polysilicon layer corresponds to the bottom backplate, which is composed of the joint of MMPOLY1 and MMPOLY2 layers of the SUMMiT V process. The diaphragm and top backplate are formed by MMPOLY3 and MMPOLY4 layers. In addition, the MMPOLY0 layer is used for the electrical connections, as shown in Figure 3b,c.

### 2.2. Diaphragm Model

**Figure 4:**
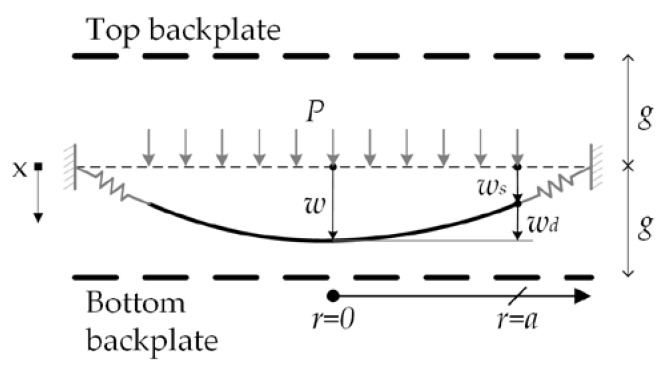
Deflection of the microphone diaphragm.

**Figure 5:**
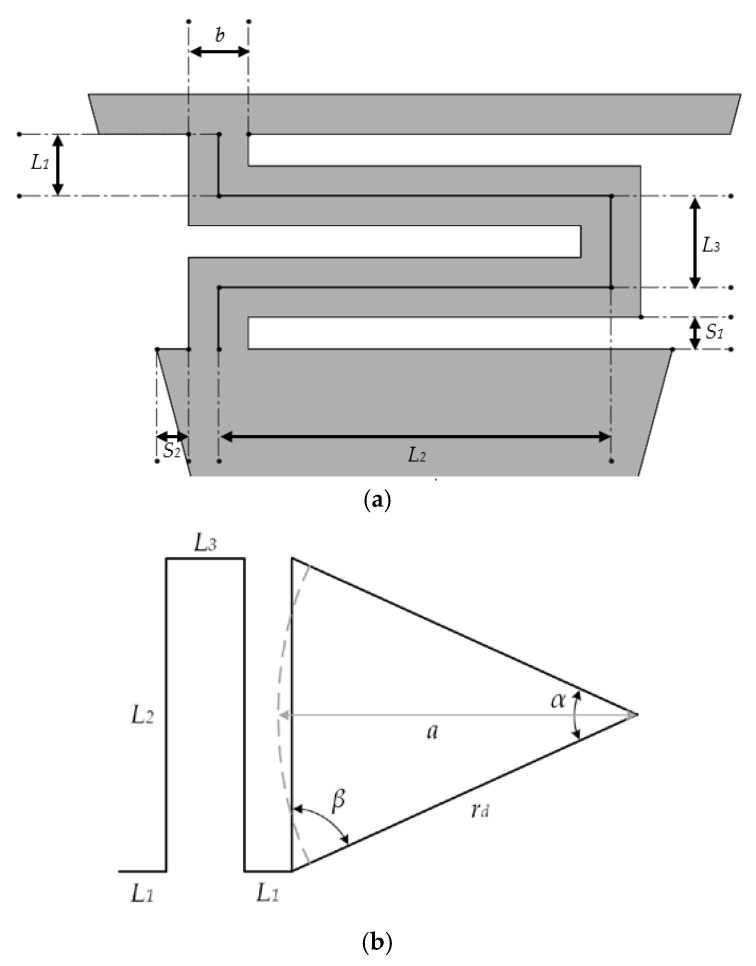
(**a**) Geometrical parameters of the polysilicon spring and (**b**) schematic representation of a partial section of the out surface of the microphone diaphragm.

The diaphragm total deflection (*w*) is calculated assuming the sum of deflections of the springs (*w_s_*) and diaphragm (*w_d_*) (see Figure 4). The interaction at the joint between the springs and diaphragm is considered to have static equilibrium. We regard that diaphragm geometry is axisymmetric and its material is homogenous and linearly elastic. Figure 5 shows the geometrical parameters of a polysilicon spring and a schematic view of a partial section of the out surface of the microphone diaphragm.

The diaphragm has a polygonal shape of *n* edges with radius *r_d_*, and it can be approximated as a circle (*a* radius) with the same area of the polygonal shape:

(1) $$a=r_{d}\sqrt{\frac{n\sin\left(\alpha \right)}{2\pi }}\;$$

The dimensions of the spring (see Figure 5a) can be proposed as function of the following geometrical parameters:

(2) $$L_{1}=\frac{b}{2}+S_{1}$$

(3) $$L_{2}=2r_{d}\sin(\frac{180}{n})-b-2S_{2}\;\;$$

(4) $$L_{3}=b+S_{1}\;\;\;$$

**Figure 6:**
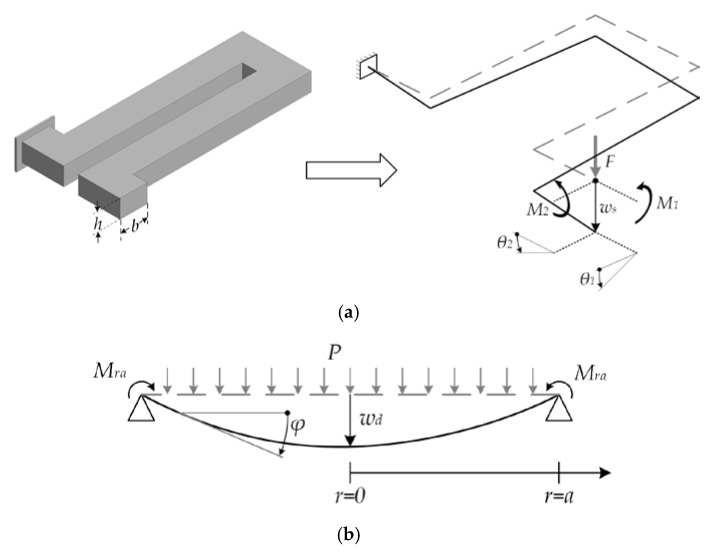
Schematic view of the deflections of the (**a**) spring and (**b**) diaphragm used in the model of the MEMS microphone.

Figure 6 shows the forces and moments on the springs and diaphragm of the microphone. The sound pressure (*P*) on the diaphragm generates the forces at the tip of the springs and diaphragm edge. The slope at the joint is equal for the springs and diaphragm. The moments *M*_1_ and *M*_2_ cause a bending moment (*M_r_*) at the diaphragm edge, which can be modeled as a moment per unit length *M_ra_*:

(5) $$F=\frac{P\pi a^{2}}{n}$$

(6) $$M_{r}=M_{1}\cos(\beta )+M_{2}\sin(\beta )\;$$

(7) $$M_{ra}=\frac{M_{r}n}{2\pi a}\;$$

(8) $$\theta_{1}=-\varphi_{a}\cos(\beta )\;$$

(9) $$\theta_{2}=-\varphi_{a}\sin(\beta )\;$$

Using Castigliano’s second theorem [15], we obtain the displacements (*w*_s_) and rotations at the spring tip:

(10) $$w_{s}=c_{1}F+c_{2}M_{1}+c_{3}M_{2}\;$$

(11) $$M_{1}=k_{1}F+k_{2}\theta_{1}\;$$

(12) $$M_{2}=k_{3}F+k_{4}\theta_{2}\;$$

with

(13) $$c_{1}=\frac{2L_{1}^{2}L_{2}}{GJ}+\frac{2L_{1}L_{3}^{2}}{EI}+\frac{4L_{1}^{2}L_{3}}{EI}+\frac{L_{2}^{2}L_{3}}{GJ}+\frac{L_{2}L_{3}^{2}}{GJ}+\frac{L_{3}^{3}}{3EI}+\frac{2L_{2}^{3}}{3EI}+\frac{2L_{1}L_{2}L_{3}}{GJ}+\frac{8L_{1}^{3}}{3EI}\;$$

(14) $$c_{2}=\frac{L_{2}L_{3}}{GJ}+\frac{L_{2}^{2}}{EI}\;$$

(15) $$c_{3}=-\frac{2L_{1}L_{2}}{GJ}-\frac{2L_{1}L_{3}}{EI}-\frac{L_{2}L_{3}}{GJ}-\frac{2L_{1}^{2}}{EI}-\frac{L_{3}^{2}}{2EI}\;$$

(16) $$k_{1}=-\frac{L_{2}\left(EIL_{3}+GJL_{2}\right)}{2EIL_{1}+EIL_{3}+2GJL_{2}}\;$$

(17) $$k_{2}=\frac{GJEI}{2EIL_{1}+EIL_{3}+2GJL_{2}}\;\;$$

(18) $$k_{3}=L_{1}+\frac{L_{3}}{2}\;\;$$

(19) $$k_{4}=\frac{EIGJ}{2EIL_{2}+2GJL_{1}+GJL_{3}}\;$$

where *L*_1_, *L*_2_, and *L*_3_ are the dimensions of the spring, *E* and *G* are the elastic and shear modulus of the polysilicon, respectively, *J* and *I* are the polar moment of inertia and moment of inertia of the spring.

*J* and *I* can be determined by [16]:

(20) $$J=\frac{bh^{3}}{3}\left(1-\frac{192h}{\pi^{5}b}\overset{\infty }{\underset{m=0}{\sum }}\frac{1}{{\left(2m+1\right)}^{5}}\tanh\left(\frac{(2n+1)\pi b}{2h}\right)\right)\;$$

(21) $$I=\frac{bh^{3}}{12}\;$$

where *b* and *h* are the width and thickness of the cross-section of the springs, respectively.

Assuming small displacements, the transverse deflection (*w_d_*) of the diaphragm can be expressed as [17]:

(22) $$w_{d}(P,r)=\;\frac{P{\left(a^{2}-r^{2}\right)}^{2}}{64D}+\eta \frac{Pa^{2}\left(a^{2}-r^{2}\right)}{16D(1+v)}\;$$

(23) $$D=\frac{Eh^{3}}{12(1-v^{2})}\;$$

where *D* is the flexural rigidity, *ν* is the Poisson’s ratio, *r* is the radial coordinate and *η* is a factor that considers the elastic support generated by the springs. 

If the springs are very flexible then *η* has the value unity, in which the diaphragm edge behaves as simply supported. If the springs are very rigid, then *η* has small values and the diaphragm edge behaves as clamped. The slope (*ϕ*) of the diaphragm deflection (see Figure 6b) is calculated as:

(24) $$\varphi =-\frac{d\left(w_{d}\right)}{dr}\;=\;\frac{Pr\left(a^{2}-r^{2}\right)}{16D}+\eta \frac{Pa^{2}r}{8D(1+v)}\;$$

(25) $$\varphi_{a}=\varphi_{\left(r=a\right)}\;=\;\eta \frac{Pa^{3}}{8D(1+v)}=\eta \frac{Fna}{8D\pi (1+v)}\;$$

The bending moment on the diaphragm edge (*M_ra_*) is determined by:

(26) $$M_{r}=D\left(\frac{d\varphi }{dr}+\frac{\nu }{r}\varphi \right)=\;\frac{P}{16}\left(2a^{2}\eta +a^{2}v-r^{2}v+a^{2}-3r^{2}\right)\;$$

(27) $$M_{ra}=M_{\left(r=a\right)}=\;\frac{Pa^{2}}{8}(\eta -1)\;$$

Using Equations (5), (11), (12) and (27), the value of *η* can be obtained by:

(28) $$\eta =\frac{2D\pi (1+v)(4k_{1}\cos\beta +4k_{3}\sin\beta +a)}{a(k_{2}n{\left(\cos\beta \right)}^{2}+k_{4}n{\left(\sin\beta \right)}^{2}+2D\pi (1+v))}\;$$

The compliance of the springs (*C_s_*) is calculated by:

(29) $$C_{s}=\frac{w_{s}}{nF}=\frac{1}{n}\left(c_{1}+c_{2}\left(k_{1}-k_{2}\eta \frac{na\cos\beta }{8D\pi (1+v)}\right)+c_{3}\left(k_{3}-k_{4}\eta \frac{na\sin\beta }{8D\pi (1+v)}\right)\right)\;$$

Finally, total deflection (*w*) of the diaphragm is determined by:

(30) $$w(P,r)=P\left(C_{s}\pi a^{2}+\;\frac{{\left(a^{2}-r^{2}\right)}^{2}}{64D}+\eta \frac{a^{2}\left(a^{2}-r^{2}\right)}{16D(1+v)}\right)\;\;$$

Equation (30) can be indicated as:

(31) $$w(P,r)=P\left(B_{1}r^{4}+B_{2}r^{2}+B_{3}\right)\;$$

with

(32) $$B_{1}=\frac{1}{64D}\;$$

(33) $$B_{2}=-\frac{a^{2}}{32D}-\frac{\eta a^{2}}{16D(1+v)}\;$$

(34) $$B_{3}=C_{s}\pi a^{2}+\frac{a^{4}}{64D}+\frac{\eta a^{4}}{16D(1+v)}\;$$

### 2.3. Mechanical Lumped Parameter Model

**Figure 7:**
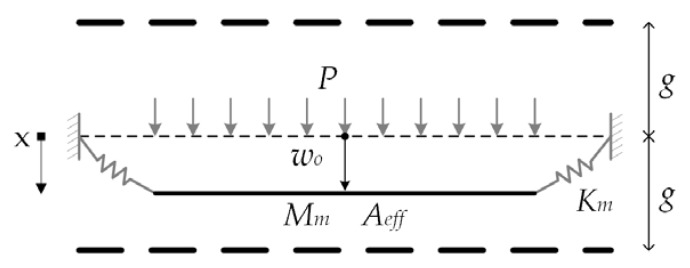
Spring and piston model of the diaphragm.

To achieve further analysis, we consider the distributed diaphragm as a piston of mass (*M_m_*) supported by a spring with stiffness (*K_m_*), as shown in Figure 7. The piston area (*A_eff_*) is used to maintain the continuity between the volumetric flow rate of the distributed diaphragm and the lumped model. Assuming a uniform pressure, the piston deflection is equal to the deflection of the diaphragm center:

(35) $$w_{0}=\frac{PA_{eff}}{K_{m}}\;$$

The potential and kinetic energies of the deformed diaphragm are represented by the lumped stiffness (*K_m_*) and lumped mass (*M_m_*), respectively. The effective area ensures the same value of the volumetric flow and it is equal to the area of the diaphragm multiplied by a factor called relative area (*A_r_*) [18]. The detailed derivation of the lumped elements is given in Appendix A:

(36) $$K_{m}=\frac{64D\pi \left(1+v\right)\left(a^{2}\left(6\eta +1+v\right)+192C_{s}D\pi \left(1+\nu \right)\right)}{3{\left(a^{2}\left(4\eta +1+v\right)+64C_{s}D\pi \left(1+v\right)\right)}^{2}}\;$$

(37) $$M_{m}=\rho h\pi a^{2}\left(\frac{6B_{1}^{2}a^{8}+15B_{1}B_{2}a^{6}+10a^{4}(2B_{1}B_{3}+B_{2}^{2})+30B_{2}B_{3}a^{2}+30B_{3}^{2}}{30B_{3}^{2}}\right)\;$$

(38) $$A_{eff}=\pi a^{2}A_{r}\;\;$$

(39) $$A_{r}=\frac{a^{2}(v+6\eta +1)+192C_{s}D\pi (1+v)}{3a^{2}(v+4\eta +1)+192C_{s}D\pi (1+v)}\;$$

### 2.4. Electrical Model

**Figure 8:**
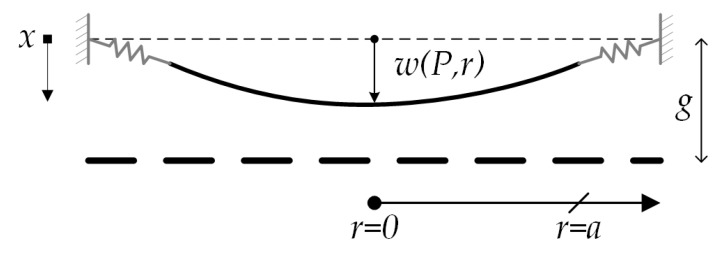
Model of the microphone capacitor.

Figure 8 depicts a model of the microphone capacitance, which is used to convert the sound pressure into an electrical signal. The microphone total capacitance (*C*) can be determined by [7]:

(40) $$C=\int_{0}^{a}\frac{2\pi \varepsilon_{0}rdr}{g-w(P,r)}\;$$

By substituting Equation (31) in (40), we obtain:

(41) $$C=\;-\frac{2\pi \varepsilon_{0}}{B_{4}}\left(\tan^{-1}\left(\frac{P\left(2B_{1}a^{2}+B_{2}\right)}{B_{4}}\right)-\tan^{-1}\left(\frac{PB_{2}}{B_{4}}\right)\right)\;$$

with

(42) $$B_{4}=\sqrt{\left(4B_{1}B_{3}-B_{2}^{2}\right)P^{2}-4gB_{1}P}\;$$

A linear approximation of the microphone capacitance without considering the blackplate holes is obtained using a Taylor series expansion:

(43) $$C=\frac{\varepsilon_{0}\pi a^{2}}{g}+\frac{\varepsilon_{0}\pi a^{2}\left(2B_{1}a^{4}+3B_{2}a^{2}+6B_{3}\right)P}{6g^{2}}=C_{0}+\Delta C\;$$

In Equation (43), the first term is the mean capacitance and the second term is the capacitance shift as function of the incident sound pressure. To take into account the effect of the backplates holes in the capacitance, we introduce a correction factor (*γ*). This factor is the ratio of the capacitance of the backplate with holes to the pure plate capacitance and it is estimated with the commercial software ANSYS [19] in Section 3. The previous expression for the capacitance can be rewritten using lumped elements and the correction factor (*γ*) as:

(44) $$C_{0}=\gamma \frac{\varepsilon_{0}\pi a^{2}}{g}\;$$

(45) $$\Delta C=\frac{PC_{0}\pi a^{2}A_{r}^{2}}{gK_{m}}=\frac{w_{0}C_{0}A_{r}}{g}\;$$

**Figure 9:**
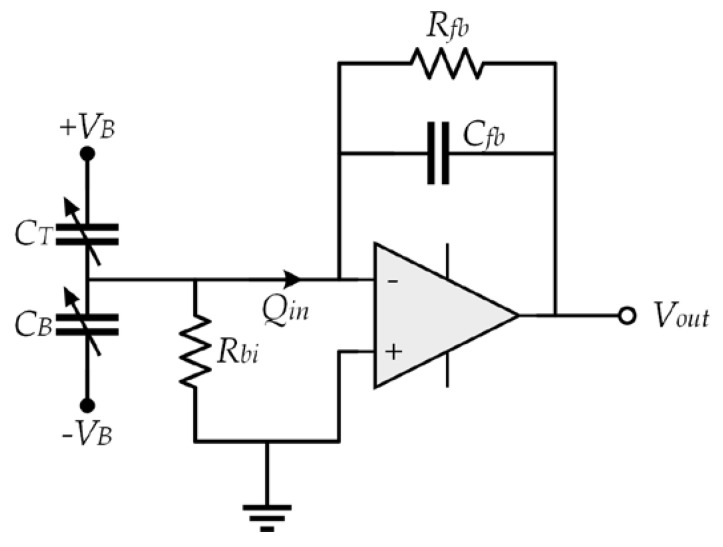
Model of the deformed capacitor.

By applying a constant bias voltage to the top and bottom capacitor and using a charge amplifier, it is possible to transform the capacitance shift into an output voltage. Figure 9 depicts a schematic of the charge amplifier, where the varying component of the charge on the capacitors (*Q_in_*) is stored in the feedback capacitor to generate an output voltage.

The charges on the top (*Q_T_*) and bottom (*Q_B_*) capacitor are obtained as:

(46) $$Q_{T}=V_{B}C_{T}=V_{B}\left(C_{T0}+\Delta C_{T}\right)\;$$

(47) $$Q_{B}=V_{B}C_{B}=-V_{B}\left(C_{B0}-\Delta C_{B}\right)\;$$

where *V_B_* is the bias voltage, *R_fb_* is the feedback resistance, *R_bi_* is the bias resistance, *C_T_*_0_ and *C_B_*_0_ are the mean capacitance of the top and bottom capacitor, respectively, and Δ*C_T_* and Δ*C_B_* are the capacitance shifts of the top and bottom capacitor, respectively.

The charge variation (*Q_in_*) of both (top and bottom) capacitors is obtained as:

(48) $$Q_{in}=\Delta Q_{T}+\Delta Q_{B}=V_{B}\left(\Delta C_{T}+\Delta C_{B}\right)\;$$

The output voltage (*V_out_*) of the operational amplifier depends on the feedback capacitor *C_fb_*:

(49) $$V_{out}=\frac{Q_{in}}{C_{fb}}=\frac{V_{B}\left(\Delta C_{T}+\Delta C_{B}\right)}{C_{fb}}\;$$

The bias voltage supplied to microphone plates generates an electrostatic force on the diaphragm. This force is opposite to restoring force of the diaphragm and it can cause instability of the diaphragm when the bias voltage largely increases, collapsing the diaphragm. In a quasi-static analysis and considering the sound pressure, we obtain the following equation of forces on the diaphragm:

(50) $$\frac{\varepsilon_{0}A_{eff}V^{2}}{2{\left(g+x\right)}^{2}}-\frac{\varepsilon_{0}A_{eff}V^{2}}{2{\left(g-x\right)}^{2}}-PA_{eff}+K_{m}x=0\;$$

where *x* is the deflection of the diaphragm, *A_eff_* is the effective area and *K_m_* is the lumped stiffness.

The pull-in phenomenon of the diaphragm will occur when the derivate of the voltage with respect to position is zero [20]. Thus, pull-in voltage (*V_PI_*) of the diaphragm can be approximated by:

(51) $$V_{PI}=\sqrt{\frac{\left(K_{m}x_{PI}-PA_{eff}\right){\left(g^{2}-x_{PI}^{2}\right)}^{2}}{2\varepsilon_{o}A_{eff}gx_{PI}}}\;$$

with

(52) $$x_{PI}=\frac{\sqrt[{3}]{f_{0}}}{4K_{m}}+\frac{{\left(PA_{eff}\right)}^{2}}{4K_{m}\sqrt[{3}]{f_{0}}}+\frac{PA_{eff}}{4K_{m}}\;$$

(53) $$\;f_{0}=PA_{eff}\left(8g^{2}K_{m}^{2}+4gK_{m}\sqrt{4g^{2}K_{m}^{2}+{\left(PA_{eff}\right)}^{2}}+{\left(PA_{eff}\right)}^{2}\right)\;$$

If there is no pressure on the diaphragm, the maximum pull-in voltage (*V_MPI_*) is given by:

(54) $$V_{MPI}=\sqrt{\frac{g^{3}K_{m}}{2\varepsilon_{0}A_{eff}}}\;$$

The electrostatic force (*F_es_*) generates another effect at normal operation conditions of the microphone due to its opposite direction, named the electrostatic spring softening. This effect has a behavior similar to a negative stiffness (*K_es_*) and it can be estimated using the linear term of the Taylor series of the total electrostatic force [20]:

(55) $$F_{es}=\frac{\varepsilon_{0}A_{eff}V_{b}{}^{2}}{2{\left(g+x\right)}^{2}}-\frac{\varepsilon_{0}A_{eff}V_{b}{}^{2}}{2{\left(g-x\right)}^{2}}\approx -\frac{2\varepsilon_{0}A_{eff}V_{b}{}^{2}}{g^{3}}x-\frac{4\varepsilon_{0}A_{eff}V_{b}{}^{2}}{g^{5}}x^{3}\;$$

(56) $$K_{es}=\frac{2\varepsilon_{0}AV_{b}{}^{2}}{g^{3}}\;$$

### 2.5. Damping Model

**Figure 10:**
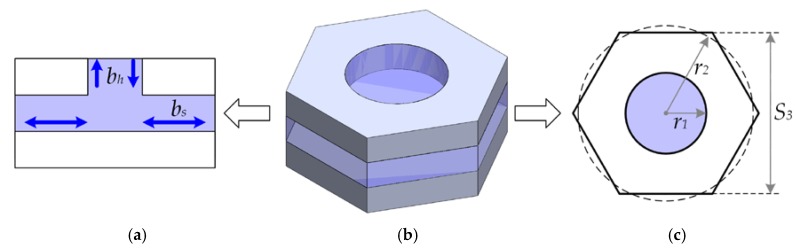
(**a**) Cross-section view of a blackplate hole model with squeeze-film damping and damping through hole; (**b**) 3D view of the blackplate hole model and (**c**) its dimensions.

There are two main sources of viscous damping on the microphone: The air flow between the backplates and diaphragm, and the air flow between springs. In the first source (see Figure 10), there are two effects that generate the viscous damping: The horizontal air flow between the plates known as squeeze-film damping (*b_s_*) and the air flow through the holes (*b_h_*). 

We use the minimum total damping coefficient (*b_m_*) given by [21], which is determined in Appendix B:

(57) $$b_{m}=b_{s}+b_{h}=\pi a^{2}\frac{8\mu \sqrt{6}}{A_{RH}}\sqrt{\frac{h}{g^{3}}\left(\frac{A_{RH}}{2}-\frac{A_{RH}^{2}}{8}-\frac{1}{4}\ln\left(A_{RH}\right)-\frac{3}{8}\right)}\;$$

where *µ* is the viscosity of air and *A_RH_* is the ratio of the hole area to the total area.

**Figure 11:**
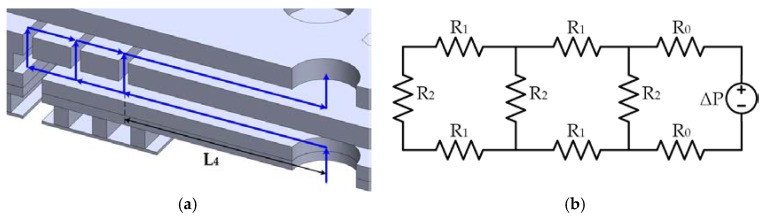
(**a**) Acoustic resistance and (**b**) equivalent hydraulic circuit of the MEMS microphone.

The space between the springs operates as a vent that connects the environment with the back cavity and generates a pressure drop, as shown in Figure 11. This damping source is analyzed using acoustic resistance, the ratio of the pressure to the volumetric flow.

By assuming a laminar flow inside of the slits, space between the backplates and diaphragm, the acoustic resistance (*R_eq_*) of one spring can be estimated using an equivalent hydraulic circuit.

(58) $$R_{eq}=2R_{0}+\frac{R_{2}\left(4R_{1}^{2}+6R_{1}R_{2}+R_{2}^{2}\right)}{4R_{1}^{2}+8R_{1}R_{2}+3R_{2}^{2}}\;$$

where *R*_0_, *R*_1_, *R*_2_ are the acoustic resistance of the slits given by [21]:

(59) $$R_{0}=\frac{12\mu L_{4}}{g^{3}L_{2}}\;$$

(60) $$R_{1}=\frac{12\mu b}{g^{3}L_{2}}\;$$

(61) $$R_{2}=\frac{12\mu h}{S_{1}^{3}L_{2}}\;$$

Due to *n* springs in the equivalent hydraulic circuit, the total acoustic resistance can be determined as:

(62) $$R_{a,s}=\frac{1}{n}\left(2R_{0}+\frac{R_{2}\left(4R_{1}^{2}+6R_{1}R_{2}+R_{2}^{2}\right)}{4R_{1}^{2}+8R_{1}R_{2}+3R_{2}^{2}}\right)\;$$

### 2.6. Lumped Element Modeling

To determine the dynamic behavior of the microphone, we use lumped elements considering the acoustic energy domain and coupling with the electrical domain through ideal transformers. The acoustic lumped parameters are estimated through mechanical lumped parameters with the following relation [22,23]:

(63) $$Z_{a}=\frac{Z_{m}}{A_{eff}^{2}}\;$$

where *Z_m_* is the mechanical impedance (i.e., the ratio of the force to the velocity), *Z_a_* is the acoustic impedance (i.e., the ratio of the pressure to the volumetric flow) and *A_eff_* is the effective area of the diaphragm. The lumped elements are represented by electrical components, as shown in Table 1.

**Figure 12:**
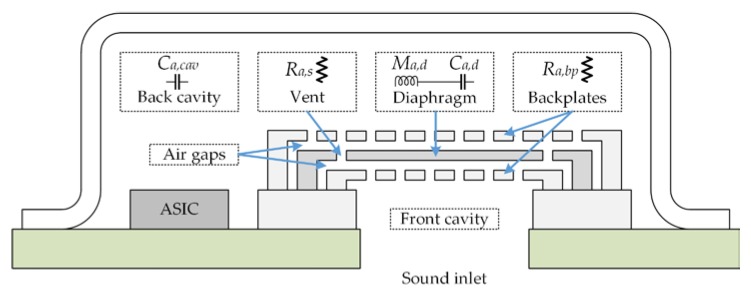
Elements of the electroacoustic lumped model of the MEMS microphone.

**Figure 13:**
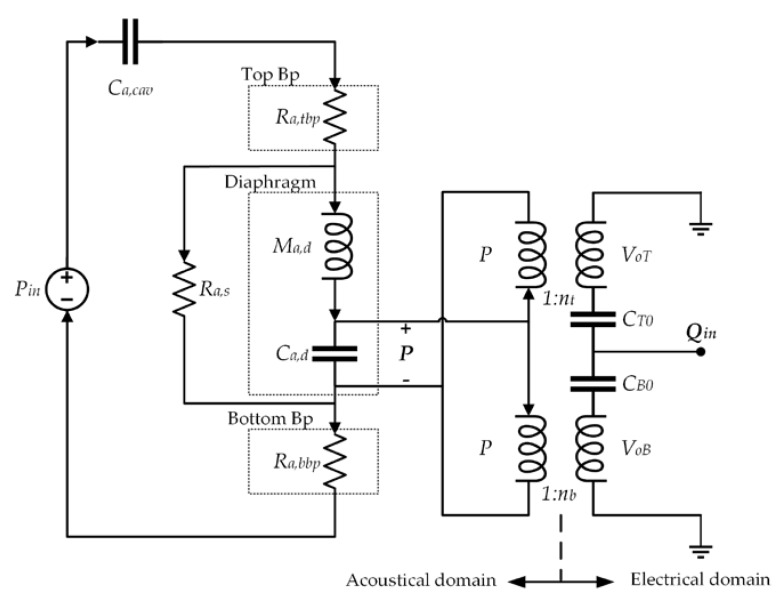
Electroacoustic lumped model of the MEMS microphone.

Figure 12 and Figure 13 show the lumped elements and the simplified electroacoustic lumped model of the microphone, respectively. To simplify the model, we consider the backplates to be rigid and neglect their compliance. In addition, the effect of the bottom port is neglected, as well as the compliance of the air gaps between the backplates and diaphragm. The incident pressure (*P_in_*) flows through the backplates holes, causing a pressure drop (*P*) that deflects the diaphragm. The diaphragm deflection is converted into an output charge using ideal transformers.

Table 2 depicts the elements used in the electroacoustic lumped model of the microphone. A lumped compliance (capacitor) represents the storage of potential energy, a lumped mass (inductance) indicates the storage of kinetic energy and a lumped resistance represents the dissipation of energy due to damping forces. Moreover, an effective area ensures that volumetric flow is equal to that of the lumped model and it is used to relate the mechanical domain with the acoustic domain.

Taking into account the electrostatic spring softening (Equation (49)), the total mechanical compliance (*C_m_*) of the diaphragm is determined as:

(64) $$C_{m}=\frac{1}{K_{m}-K_{es}}\;$$

The value of the acoustic cavity compliance (*C_a,cav_*) represent the storage of potential energy in the compressed air and is given by [24]:

(65) $$C_{a,cav}=\frac{V_{cav}}{\rho_{air}c_{0}^{2}}\;$$

where *V_cav_* is the volume of the cavity and *c*_0_ is the isentropic speed of sound at ambient temperature.

The acoustic resistances of the top and bottom backplates are considered equal and their values are determined by:

(66) $$R_{a,tbp}=R_{a,bbp}=\frac{b_{m}}{A_{eff}^{2}}\;$$

The charge shift (Δ*Q_c_*) of one capacitor at the electroacoustic model is estimated by:

(67) $$\Delta Q_{c}=C_{0}V_{0}=C_{0}nP\;$$

The previous expressions for the charge shift can be rewritten using lumped elements:

(68) $$\Delta Q_{c}=V_{B}\Delta C=P\frac{V_{B}C_{0}\pi a^{2}A_{r}^{2}}{g\left(K_{m}-K_{es}\right)}\;$$

We assume that the turn ratio is equal for both capacitors and it can be deduced from the previous equation:

(69) $$n_{t}=n_{b}=\frac{V_{B}\pi a^{2}A_{r}^{2}}{g\left(K_{m}-K_{es}\right)}\;$$

The frequency response of the microphone is obtained by the transfer function of the equivalent model circuit (see Figure 13):

(70) $$H_{mic}=\frac{P}{P_{in}}=\frac{sC_{a,cav}R_{a,s}}{H_{1}s^{3}+H_{2}s^{2}+H_{3}s+1}\;$$

with

(71) $$H_{1}=C_{a,d}M_{a,d}C_{a,cav}\left(R_{a,tbp}+R_{a,bbp}+R_{a,s}\right)\;$$

(72) $$H_{2}=C_{a,d}\left(C_{a,cav}R_{a,s}\left(R_{a,tbp}+R_{a,bbp}\right)+M_{a,d}\right)\;$$

(73) $$H_{3}=\left(C_{a,d}R_{a,s}+C_{a,cav}\left(R_{a,tbp}+R_{a,bbp}+R_{a,s}\right)\right)\;$$

The output voltage can be expressed with the previous expressions as:

(74) $$V_{out}=P_{in}\frac{\left(C_{T0}n_{t}+C_{B0}n_{b}\right)H_{mic}}{C_{fb}}\;$$

The sensitivity is the ratio of the output voltage to the incident pressure on the microphone:

(75) $$S=S_{d}H_{mic}\;$$

(76) $$S_{d}=\frac{2}{C_{fb}}\left(\frac{V_{B}C_{0}\pi a^{2}A_{r}^{2}}{g\left(K_{m}-K_{es}\right)}\right)\;$$

where *S_d_* is the sensitivity of the microphone diaphragm, the ratio of the output voltage to the pressure on the diaphragm.

**Figure 14:**
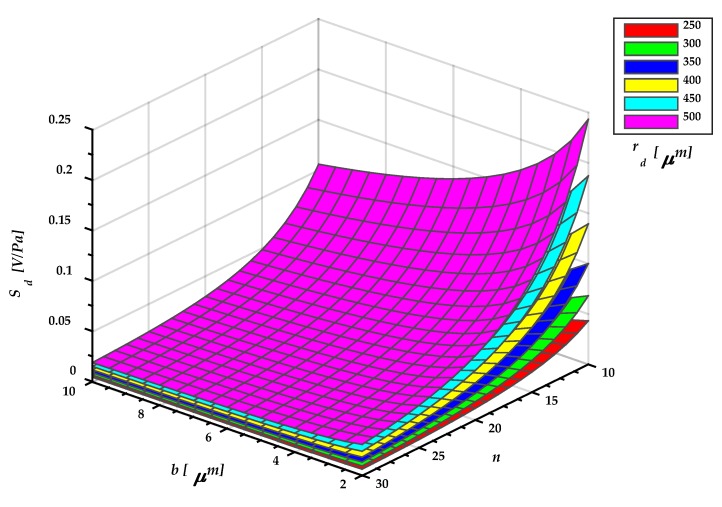
Sensitivity of the MEMS microphone diaphragm.

Figure 14 shows the sensitivity of the microphone diaphragm as function of geometrical parameters: Width of the springs (*b*), number of springs (*n*) and the radius of the polygonal diaphragm (*r_d_*). With our design is possible to modify the sensitivity for a diaphragm with constant radius, something that would be difficult with a clamped or simple supported diaphragm. To obtain this graphic, we consider that the bias voltage is 30% of the value of the maximum pull-in voltage given by Equation (54) and the feedback capacitance has the same value of initial capacitance *C*_0_.

### 2.7. Noise Model

The thermomechanical noise and electrical noise determine the minimum sound pressure that the microphone can measure. The signal-to-noise ratio (SNR) is a useful value to determine the performance of a microphone and it is calculated by [4]:

(77) $$SNR=94dB-P_{\min}\;$$

where *P*_min_ is the minimum pressure (decibels units) employing a A-weighted filter to take into account the behavior of the human ear.

#### 2.7.1. Acoustic Noise

The acoustic resistance generates a random noise that is proportional to temperature (*T_R_*) and it can be modeled using a pressure or volumetric noise source. The noise due to resistance *R_eff_* is obtained using a pressure noise source (*S_np_*_,*Reff*_). In addition, the noise due to *R_a_*_,*s*_ is estimated with a volumetric noise source (*S_nq_*_,*Ras*_). Also, the power spectrum densities (PSD) of these noise sources are calculated as [25]:

(78) Snp,Reff=4kBTRRReff 

(79) $$S_{nq,Ras}=\frac{4k_{B}T_{R}}{R_{a,s}}\;$$

where *k_B_* is Boltzmann’s constant and *R_eff_* is the sum of the acoustic resistance of the top and bottom plate.

**Figure 15:**
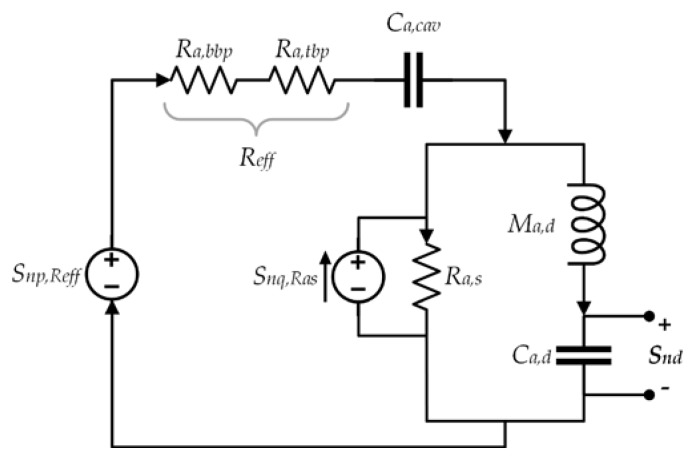
Thermomechanical noise model of the MEMS microphone.

Figure 15 depicts a lumped model of the microphone including noise sources, in which each resistor generates a noise and the total noise PSD of the diaphragm (*S_nd_*) is the sum of the two noise sources [25]:

(80) $$S_{nd}={\left|H_{n,Reff}\left(j2\pi f\right)\right|}^{2}S_{np,Reff}+{\left|H_{n,Ras}\left(j2\pi f\right)\right|}^{2}S_{nq,Ras}\;$$

(81) $$H_{n,Reff}=H_{mic}\;$$

(82) $$H_{n,Ras}=\frac{sC_{a,cav}R_{a,s}\left(R_{a,bbp}+R_{a,tbp}\right)+R_{a,s}}{H_{1}s^{3}+H_{2}s^{2}+H_{3}s+1}\;$$

where *H_n,Reff_*, and *H_n,Ras_* are the transfer functions of the noise sources. 

#### 2.7.2. Electrical Noise

**Figure 16:**
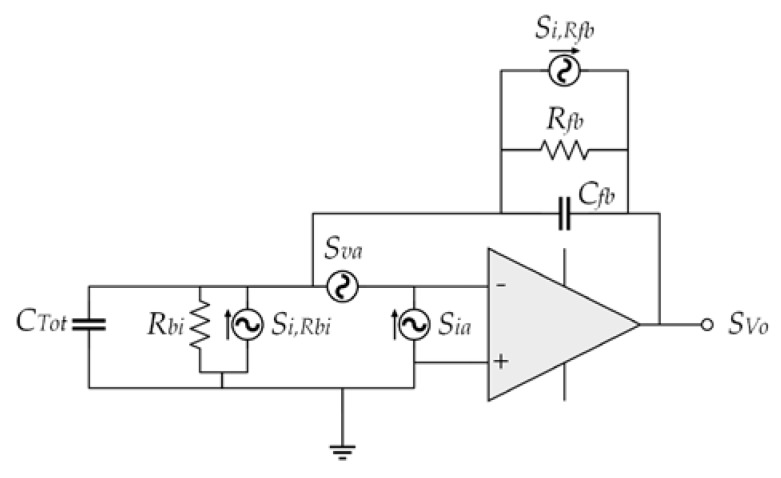
Electrical noise model of the charge amplifier.

Figure 16 depicts the noise model of the charge amplifier with their noise sources. *C_Tot_* is the total capacitance of the microphone, including parasitic capacitance, the noise generated for the resistances *R_fb_* and *R_bi_* are modeled with current sources. The internal noise of the amplifier is calculated using a voltage and a current noise source (*S_va_* and *S_ia_*), respectively. These values are obtained of the amplifier.

The PSD of the electrical noise is the sum of the noise sources considering the amplifier behavior, which can be predict by [7]:

(83) $$S_{Vo}=S_{va}{\left|1+\frac{Z_{fb}}{Z_{i}}\right|}^{2}+{\left|Z_{fb}\right|}^{2}\left(S_{ia}+S_{i,Rfb}+S_{i,Rbi}\right)\;$$

with

(84) $$S_{i,Rfb}=\frac{4k_{B}T_{R}}{R_{fb}}\;$$

(85) $$S_{i,Rbi}=\frac{4k_{B}T_{R}}{R_{bi}}\;$$

(86) $$Z_{fb}=\frac{R_{fb}}{1+sR_{fb}C_{fb}}\;$$

(87) $$Z_{i}=\frac{R_{bi}}{1+sR_{bi}C_{Tot}}\;$$

where *Z_fb_* is the feedback impedance and *Z_i_* is the input impedance.

The acoustic noise is converted into an electrical noise knowing the diaphragm sensitivity (*S_d_*). The total noise PSD at the amplifier output is the sum of the acoustic and electrical noise. The mean square noise at the amplifier output is given by the integral of the total noise PSD multiplied by the A-weighted filter *R_Af_*:

(88) $$\bar{V_{n}^{2}}=\int_{f_{1}}^{f_{2}}\left(S_{nd}(f)S_{d}^{2}+S_{Vo}(f)\right)R_{Af}^{2}(f)df\;$$

where *f*_1_ and *f*_2_ are 20 Hz and 20 kHz, respectively.

The minimum pressure detected by the microphone is calculated dividing the root mean square of the output voltage noise respect to the sensitivity (*S*):

(89) $$P_{\min}=\frac{\bar{V_{n}}}{S}\;$$

### 2.8. Microphone Design

The microphone design can be modified to achieve different requirements such as the resonant frequency or sensitivity. The resonant frequency of the diaphragm has a direct impact on the behavior of the microphone at high frequencies and it can be approximated as [26]:

(90) $$f_{n}=\frac{1}{2\pi }\sqrt{\frac{K_{m}}{M_{m}}}\;$$

We used the design rules of the SUMMiT V fabrication process, in which the mechanical properties and dimensions of the thickness (*h*) of each polysilicon layer and the gap distance (*g*) are parameters determined by this fabrication process. For instance, we consider the resonant frequency of the microphone diaphragm as a design restriction. The configuration of the microphone diaphragm allows multiple design options to achieve different resonant frequency restrictions of the diaphragm, as shown in Table 3. 

Table 4 shows the dimensions of a MEMS microphone suitable for mobile devices. The backplate holes are distributed in a hexagonal pattern with a radius of 3.4 µm and a separation of 13 µm.

**Figure 17:**
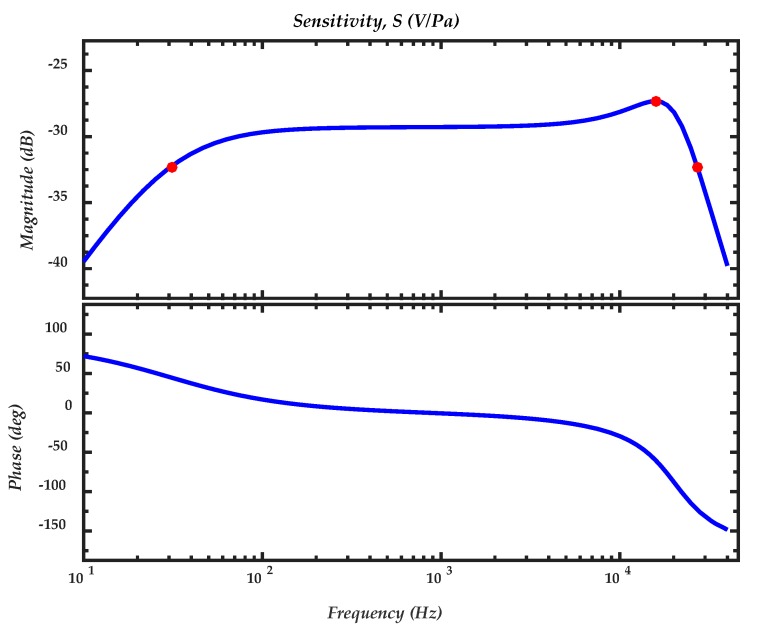
Theoretical frequency response of the MEMS microphone. The first (31 Hz) and third (27 kHz) red dots represent the bandwidth, and the second (15.8 kHz) red dot indicates the resonant frequency of the microphone.

Table 5 indicates the values of the acoustic lumped elements. Figure 17 shows the theoretical frequency response of the microphone obtained with the transfer function, Equation (70), in which is considering a bias voltage of 3 V. The microphone bandwidth is between 31 Hz to 27 kHz with a 3 dB variation. The damped resonant frequency (15.8 kHz) of the microphone using the acoustic lumped elements is smaller than that of the diaphragm (21.6 kHz). It is due to the electrostatic spring effect and the dynamic response of the microphone, which includes the damping and the back cavity effects. The sensitivity is 34.4 mV/Pa at 1 kHz and the relative area (*A_r_*) is 0.8685, which presents an improvement in comparison to a fixed supported diaphragm (0.3333) or a simply supported diaphragm (0.4608).

## 3. FEM Models

FEM models are developed to estimate the mechanical response of the MEMS microphone diaphragm considering the fifteen springs and the fringe effect of the holes discussed in Section 2.4. 

### 3.1. Electromechanical Model

These models are obtained through ANSYS Workbench software using the solid186 elements to mesh the diaphragm and the electrostatic force is coupled using tran126 and surf154. The physics properties for the FEMS models are the follows: Young modulus of 160 GPa, Poisson ratio of 0.23 and density 2330 kg/m^3^. These FEM models can estimate the deflections and the first four vibration modes of the microphone diaphragm.

**Figure 18:**
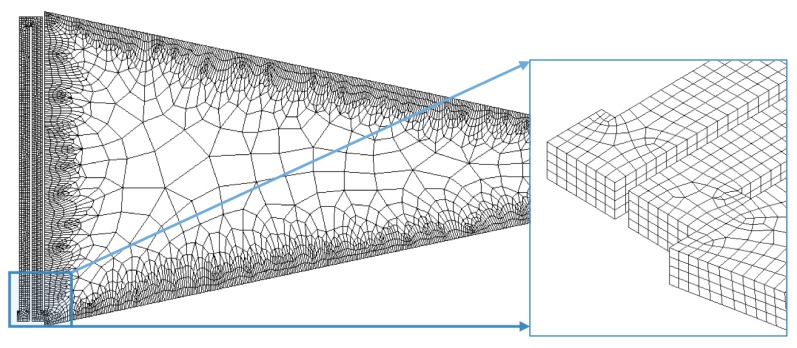
Mesh of the FEM model of the microphone diaphragm obtained through ANSYS Workbench software.

**Figure 19:**
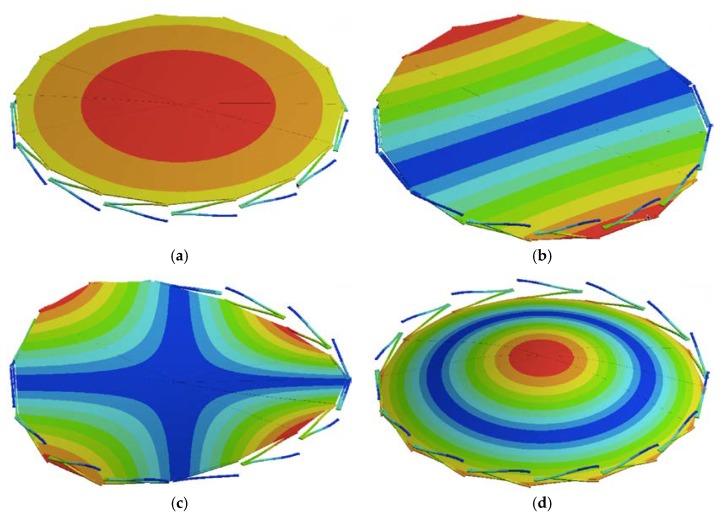
The first four vibration modes of the FEM model of the microphone diaphragm: (**a**) *f*_1_ = 21.657 kHz; (**b**) *f*_2_ = 32.891 kHz; (**c**) *f*_3_ = 68.232 kHz and (**d**) *f*_4_ = 94.674 kHz. The red and blue surfaces represent the maximum and minimum displacements, respectively.

**Figure 20:**
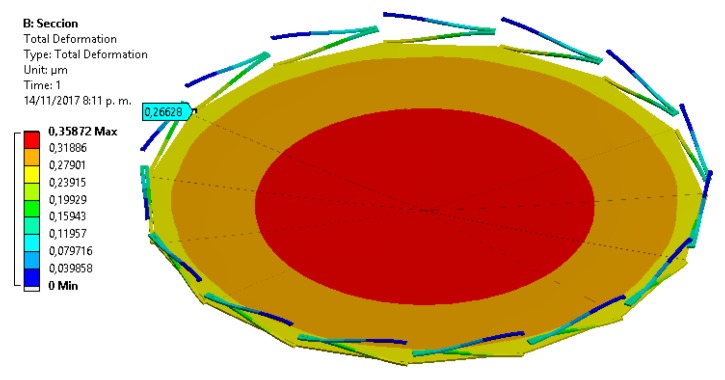
Displacements of the FEM model of the microphone diaphragm caused by a sound pressure of 30 Pa.

**Figure 21:**
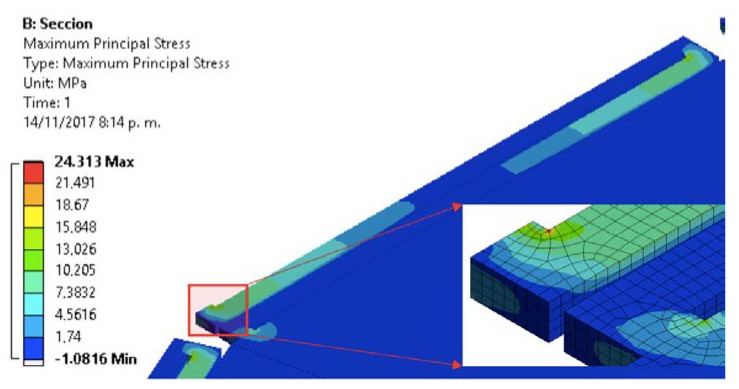
Maximum principal stress on the support springs and microphone diaphragm caused by a sound pressure of 30 Pa.

In order to simplify the FEM model, we use a symmetrical section of the diaphragm limited by an angle of 24°. Figure 18 and Figure 19 show the mesh employed in the FEM model and its first four vibration modes, the first mode (21.657 kHz) presents a relative difference of 0.44% with respect to the analytical model (21.563 kHz), and the other vibration modes are high enough to avoid interference with the normal performance of the microphone. Figure 20 shows the results of the static analysis of the microphone diaphragm under an incident sound pressure of 30 Pa. The maximum displacement of the diaphragm occurs at the center of the diaphragm, in which the springs contribute to 74% of the total displacement. Figure 21 shows the maximum principal stress (24.3 MPa) located on the clamped end of the spring. This value is less than the rupture stress (1 GPa) of the polysilicon, which is suitable for a safe operation of the microphone structure.

### 3.2. Capacitance Model

**Figure 22:**
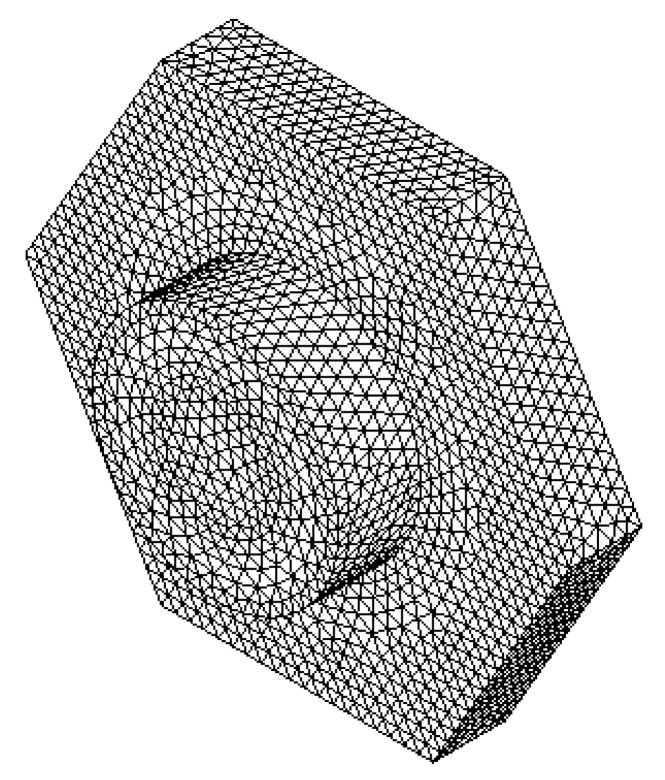
Mesh of a module formed by a hole and a section of the backplate, which is obtained through ANSYS APDL software.

**Figure 23:**
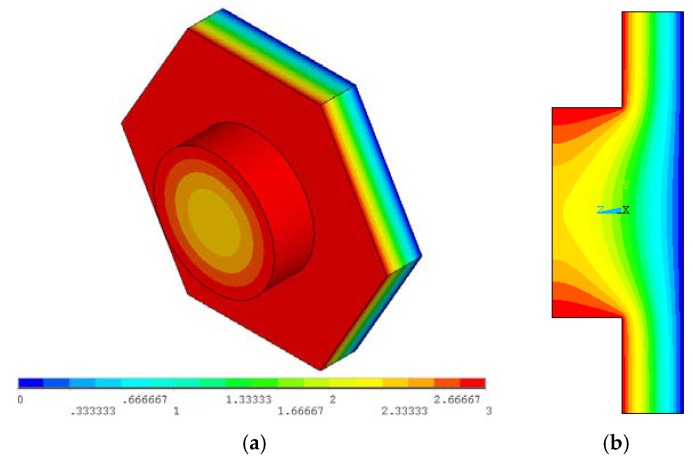
Electrical potential distribution of the proposed module: (**a**) 3D view and (**b**) cross-section view.

In order to calculate the correction factor (*γ*) of the capacitance of blackplates with holes, we take advantage of blackplate geometry that contains holes distributed uniformly on its surface area. Thus, we can analyze one module [19] formed by a hole and a section of the backplate. This module can be meshed using the finite element method (FEM) with ANSYS APDL, as shown in Figure 22. The capacitance of the module is obtained based on the storage of electrostatic energy. Figure 23 depicts the electrical potential of this module, in which the fringe effect acts on the module center. The area of the hole is 25% of the total area of the module and the correction factor (γ) is determined as 0.93, which means a capacitance reduction of 7% with respect to the pure plate capacitance.

## 4. Results and Discussion

This section presents the deflections of the FEM model of the microphone diaphragm as a function of the pressure. In addition, the results of the noise are presented and compared respect to commercial microphones and previous works reported in the literature.

**Figure 24:**
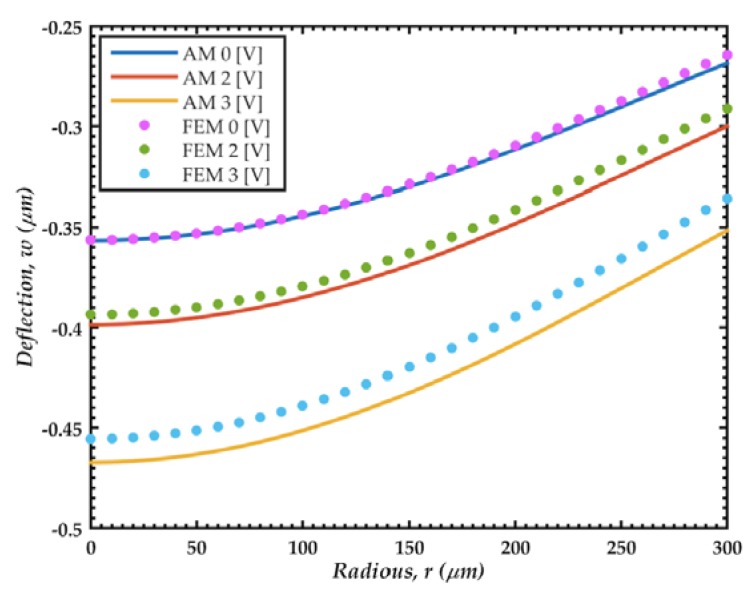
Deflections of the microphone diaphragm caused by a sound pressure of 30 Pa. These deflections are determined using analytical (AM) and FEM models.

Figure 24 shows the deflections of the microphone diaphragm under a sound pressure of 30 Pa pressure, which are obtained using the analytical and FEM models. The results of the FEM models agree well with respect to those of the analytical model. The difference presented in the shape could be due to the simplification of the electrostatic forces since the error increases with the voltage, as shown in Table 6.

**Figure 25:**
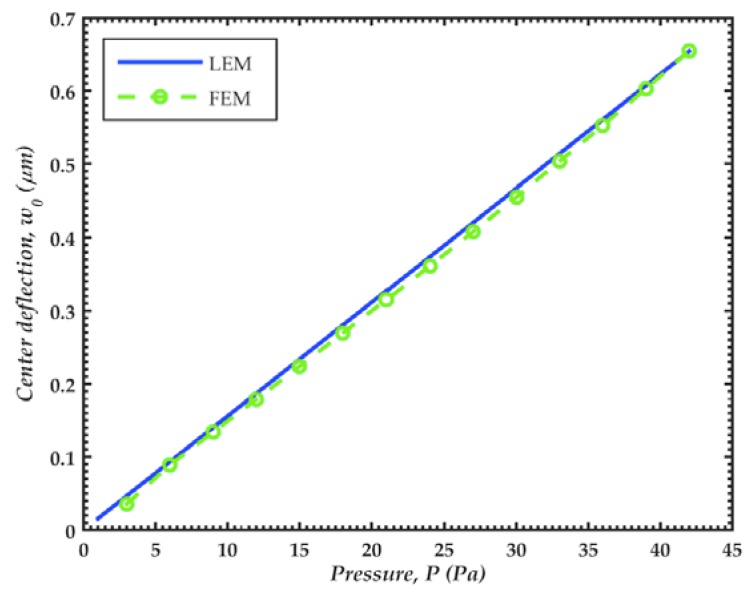
Deflections (*w*_0_) of the microphone diaphragm center as a function of the sound pressure. These deflections are calculated with a bias voltage of 3 V and using the lumped element (LEM) and FEM models.

**Figure 26:**
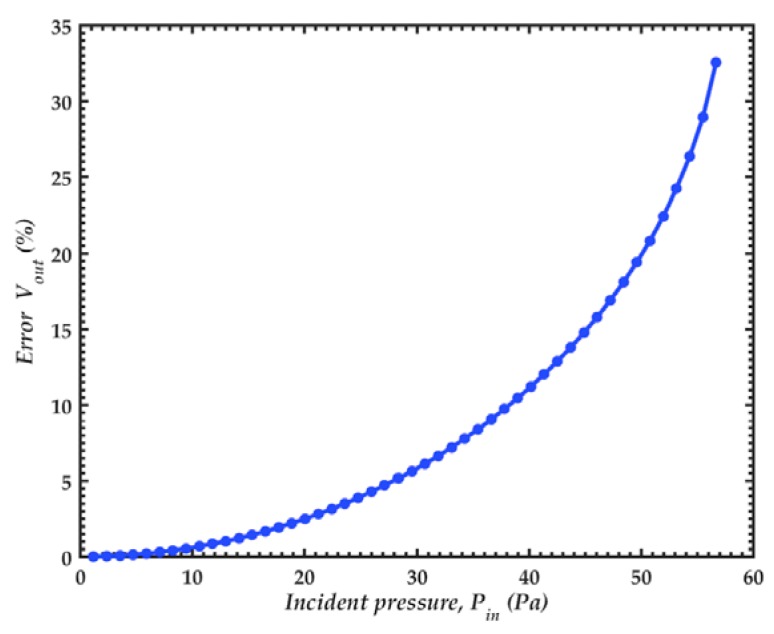
Relative error of the output voltage with a bias voltage of 3 V.

The deflection of the diaphragm center of the FEM model presents a linear behavior, as shown in Figure 25. Values of this deflection agree well with those calculated through the lumped element model (LEM). Figure 26 shows the relative error of the output voltage between the exact solution and linear approximation of the capacitance and electrostatic force of the microphone, determined by Equations (41) and (55). This relative error exponentially increases with the pressure. We assume a maximum relative error of 5% for a normal operation of the microphone. With this value, the maximum pressure of the microphone is 28 Pa peak or 20 Pa rms (120 dB). It is also observed that the pull-in phenomenon occurs with a pressure of 57 Pa.

The theoretical noise of the microphone is obtained considering the charge amplifier MAX4475, which is manufactured by Maxim Integrated^TM^ (San Jose, CA, USA) [27]. We use the values of 20 GΩ and 10 GΩ for the feedback resistor and input resistor, respectively. On the other hand, the feedback capacitance and total capacitance are 1.21 pF and 4.86 pF, respectively. This total capacitance is calculated assuming the parasitic capacitance is equal to the microphone capacitance. In addition, the input voltage-noise and input current noise are 4.5 nV/$\sqrt{\mathrm{Hz}}$ and 0.5 fA/$\sqrt{\mathrm{Hz}}$, respectively.

**Figure 27:**
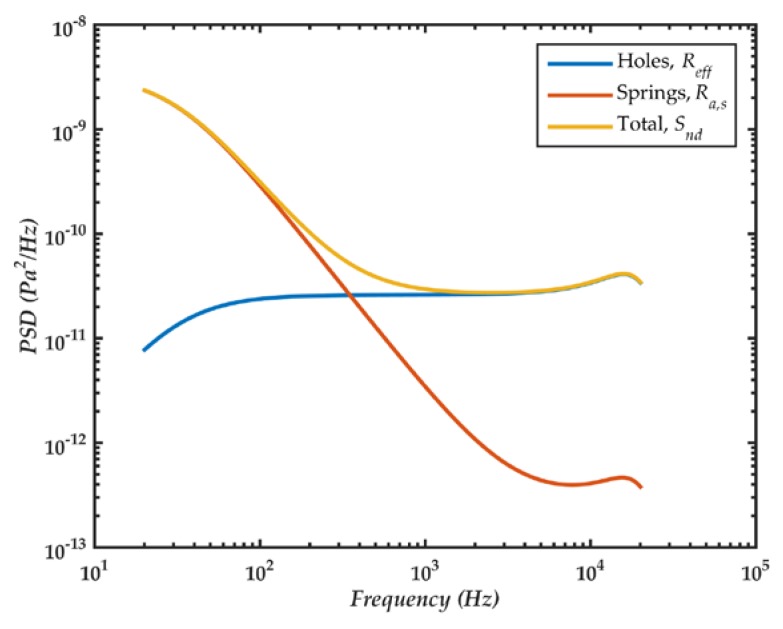
PSD of acoustic noise sources in the microphone.

Figure 27 depicts the theoretical PSD of acoustic noise sources of the MEMS microphone. The noise, generated by the slits in the springs, is proportional to 1/*f*^2^. For frequencies below 350 Hz, this noise is higher than that due to holes, which presents the same behavior as the dynamic response of the microphone. The total acoustic noise is converted to an electrical noise with the sensitivity of the diaphragm.

**Figure 28:**
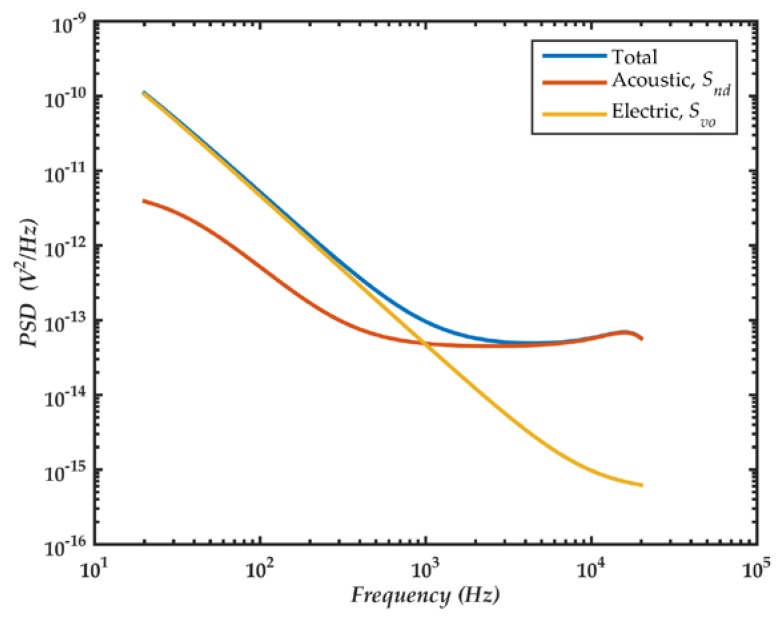
PSD of the output voltage noise in the microphone and amplifier.

Figure 28 shows the theoretical PSD of output voltage noise. The noise generated by the amplifier is proportional to 1/*f*^2^ and below 1 kHz it is higher than the acoustic noise. The minimum pressure that the microphone can detect is 820 µPa, which is obtained by applying the A-weight filter and integrating the total noise between 20 Hz and 20 kHz.

The signal to noise ratio of the proposed MEMS microphone is 61.7 dBA, which presents a good improvement in comparison with other designs: A MEMS microphone with a fixed or simply supported diaphragm of the same size and working under the same conditions would have a signal to noise ratio of 35.8 dBA and 50.8 dBA, respectively.

Table 7 depicts a summary of the theoretical properties of the proposed MEMS microphone. In addition, Table 8 shows a comparison with previous MEMS microphones. Our microphone has a sensitivity and bandwidth superior to the microphones reported in the literature [1,5,10] but has a lower SNR. The proposed design has a performance comparable with the commercial models; it was not possible to compare the internal dimensions due to the lack of information provided by the manufacturers. Our design can be improved using optimal dimensions or using a microphone array [28].

## 5. Conclusions

The novel design of a MEMS dual-backplate capacitive microphone supported by an array of fifteen polysilicon springs is presented. This design is based on SUMMiT V fabrication process from Sandia National Laboratories. The springs array allows the increment of the effective area of the diaphragm under a sound pressure, which increases the sensitivity of the microphone. Also, this springs array can modify the design parameters to obtain a specific performance of the microphone. Analytical and FEM models are developed to predict the electromechanical behavior of the microphone. The proposed microphone can operate for sound pressures within a frequency range from 31 Hz to 27 kHz. With a bias voltage of 3 V, the microphone has a sensitivity of 34.4 mV/Pa and an effective area of 86.85% respect to the total area of the diaphragm. The two backplates enable a good linear response for a maximum sound pressure of 20 Pa. The results of the analytical models agree well with those of the FEM models with a maximum error of 2.53%, which can be useful to analyze the microphone performance due to the variation of its dimensions and design parameters. The proposed microphone has characteristics suitable to be used in mobile device applications.

Future work will include the optimization of the microphone to increase the signal to noise ratio (SNR) and the fabrication of the MEMS microphone.

## Figures and Tables

**Table 1:** Lumped elements of the MEMS microphone.

Element	Electrical Equivalent	Lumped Mechanical Model	Lumped Acoustical Model
Mass	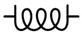	*M_m_* (kg)	*M_a_ = M_m_/A^2^_eff_* (kg/m^4^)
Spring	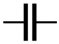	*C_m_* (m/N)	*C_a_ = C_m_A^2^_eff_* (m^5^/N)
Damper	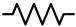	*b_m_* (N·s/m)	*R_a_ = b_m_/A^2^_eff_* (N·s/m^5^)

**Table 2:** Elements of the electroacoustic lumped model of the MEMS microphone.

Symbol	Description
*P_in_*	Incident pressure on the microphone
*R_a,s_*	Acoustic springs resistance
*C_a,cav_*	Acoustic compliance of the cavity
*R_a,tbp_*	Acoustic resistance of the top backplate
*R_a,bbp_*	Acoustic resistance of the bottom backplate
*M_a,d_*	Acoustic diaphragm mass
*C_a,d_*	Acoustic diaphragm compliance
*P*	Pressure on the diaphragm
*n_t_*	Turns ratio of top backplate
*n_b_*	Turns ratio of bottom backplate
*C_T_*_0_	Initial top capacitance
*C_B_*_0_	Initial bottom capacitance

**Table 3:** Possible designs of the MEMS microphone diaphragm.

Number of Springs, *b* = 4 µm			
*r*_0_ [µm]	*f_n_* ≈ 15 kHz	*f_n_* ≈ 20 kHz	*f_n_* ≈ 25 kHz
250	10	12	13
300	13	15	17
350	16	19	22
400	19	24	33
450	24	35	-
500	31	-	-

**Table 4:** Dimensions of the MEMS microphone.

Parameter	Value
*E*	160 GPa
*ν*	0.23
ρ	2330 kg/m^3^
*h*	2.25 µm
*g*	2 µm
*S*_1_	1 µm
*S*_2_	2 µm
*r*_0_	300 µm
*b*	4 µm
*n*	15
*L*_1_	3 µm
*L*_2_	116.75 µm
*L*_3_	5 µm
*L*_4_	18 µm
*N*	1879
*A_RH_*	25%
*V_c_*	3 mm^3^

**Table 5:** Acoustic lumped elements values.

Symbol	Value	Description
*R_a,s_*	2.0408 × 10^11^ Ns/m^5^	Acoustic springs resistance
*C_a,cav_*	2.1512 × 10^−14^ m^5^/N	Acoustic compliance of the cavity
*R_a,tbp_*, *R_a,bbp_*	1.1957 × 10^9^ Ns/m^5^	Acoustic resistance of the top and bottom backplate
*M_a,d_*	1.9219 × 10^4^ kg/m^4^	Acoustic diaphragm mass
*C_a,d_*	3.7140 × 10^−15^ m^5^/N	Acoustic diaphragm compliance
*n_t_*, *n_b_*	0.0203 V/Pa	Turns ratio top and bottom backplate
*C_T_*_0_, *C_B_*_0_	1.1304 pF	Initial top and bottom capacitance
*A_eff_*	2.3846 × 10^−7^ m^2^	Effective area
*A_r_*	0.8685	Relative area

**Table 6:** Deflections (*w*_0_) at the diaphragm center calculated through the analytical (AM) and FEM models.

	0 V	2 V	3 V
*w*_0_, AM	0.3566	0.3986	0.4673
*w*_0_, FEM	0.3564	0.3935	0.4555
Relative difference [%]	0.06	1.28	2.53

**Table 7:** Summary of the predicted properties of the MEMS microphone.

Property	Value
Sensitivity	34.4 mV/Pa (−29.3 dBV/Pa)
Signal to noise-ratio (SNR)	61.7 dBA
Bandwidth	31 Hz–27 kHz
Capacitance	2.2607 pF
Bias Voltage	3 V
Pull-in Voltage	6.17 V
Minimum pressure	820 µPa (32.1 dB)
Maximum pressure	20 Pa (120 dB)
Dynamic range	87.7 dB

**Table 8:** Comparison of the proposed design and previous MEMS microphones.

Microphone	Sensitivity [dBV/Pa]	SNR [dBA]	Bandwidth	Supply Voltage
Proposed design	−29.3	61.7	31 Hz–27 kHz	3 V
Rombach et al. (2002) [5]	−38	70	N/R–20 kHz	1.5 V
Grixti et al. (2015) [10]	−42	N/R	N/R–10.5 kHz	6 V
Kim et al. (2015) [1]	−38.4	75.8	100 Hz–20 kHz	10 V
DB Unlimited, MM034202-1	−42	58	70 Hz–16 kHz	2 V
Knowles, SPU0414HR5H-SB	−22	59	100 Hz–10 kHz	1.8 V
STMicroelectronics, MP34DT01TR-M	−26	61	100 Hz–10 kHz	1.8 V
Knowles, SPK0415HM4H-B	−26	61	100 Hz–10 kHz	3.6 V
TDK InvenSense ICS-51360	−36	62	50 Hz–20 kHz	1.8 V
Knowles, SPM0408LE5H-TB	−18	63	100 Hz–10 kHz	3.6 V
Cirrus Logic, WM7121PIMSE/RV	−38	65	200 Hz–6 kHz	3.7 V
Knowles, SPH0645LM4H-B	−26	65	10 Hz–10 kHz	3.6 V
TDK InvenSense, INMP504	−38	65	100 Hz–16 kHz	3.3 V
TDK InvenSense, INMP510	−38	65	60 Hz–20 kHz	3.3 V
TDK InvenSense ICS-40619	−36	67	20 Hz–20 kHz	2.75 V

## Appendix A

In this appendix, we present a detailed derivation of the mechanical lumped parameters for the MEMS microphone diaphragm. These parameters are used in Section 2.3.

The lumped stiffness is obtained using an analogy with a spring. The potential energy of the diaphragm is equal to that of a spring with the same deflection at the diaphragm center [19]:

(A1) $$W_{PE}=\int_{0}^{a}\int_{0}^{P_{0}}w(P,r)2\pi rdPdr\;\;$$

(A2) $$K_{m}=\frac{2W_{PE}}{\omega_{P0}^{2}}\;$$

where $W_{PE}$ is the potential energy, $K_{m}$ is the lumped stiffness and $w_{P0}$ is the deflection of the diaphragm center due to a pressure *P*_0_. By solving Equations (A1) and (A2), we obtain:

(A3) $$W_{PE}=P_{0}^{2}\left(\frac{\pi^{2}a^{4}C_{s}}{2}+\frac{\pi a^{6}}{384D}+\frac{\pi \eta a^{6}}{64D\left(1+\nu \right)}\right)\;$$

(A4) $$K_{m}=\frac{64D\pi \left(1+v\right)\left(a^{2}\left(6\eta +1+v\right)+192C_{s}D\pi \left(1+\nu \right)\right)}{3{\left(a^{2}\left(4\eta +1+v\right)+64C_{s}D\pi \left(1+v\right)\right)}^{2}}\;$$

The velocity (*v*) at any point of the diaphragm is the derivative of the diaphragm position, Equation (31), with respect to time:

(A5) $$v\left(\frac{dP}{dt},r\right)=\frac{dw(P,r)}{dt}=\left(B_{1}r^{4}+B_{2}r^{2}+B_{3}\right)\frac{dP}{dt}\;$$

The kinetic energy (*W_KE_*) of the diaphragm is obtained by integrating over the area using a differential of mass with density *ρ*:

(A6) $$W_{KE}=\frac{1}{2}{\int_{0}^{a}\left(v\left(\frac{dP}{dt},r\right)\right)}^{2}dm\;=\frac{1}{2}{\int_{0}^{a}\left(v\left(\frac{dP}{dt},r\right)\right)}^{2}2\rho h\pi rdr\;$$

In terms of a lumped mass with the same velocity of the diaphragm center (*v*_0_), we calculate:

(A7) $$W_{KE}=\frac{M_{m}v_{0}^{2}}{2}\;$$

(A8) $$M_{m}=\frac{2W_{KE}}{v_{0}^{2}}\;$$

where *M_m_* is the lumped mechanical mass of the microphone diaphragm.

By substituting Equation (A5) into (A6), *W_KE_* is determined as:

(A9) $$W_{KE}={\left(\frac{dP}{dt}\right)}^{2}\frac{\rho h\pi a^{2}}{60}\left(6B_{1}^{2}a^{8}+15B_{1}B_{2}a^{6}+10a^{4}\left(2B_{1}B_{3}+B_{2}^{2}\right)+30B_{2}B_{3}a^{2}+30B_{3}^{2}\right)\;$$

By substituting Equation (A9) into (A8), the value of the lumped mechanical mass is obtained. This mass is related to the original mass multiplied by a factor that depends on the material properties, dimensions and compliance of the springs.

(A10) $$M_{m}=\rho h\pi a^{2}\left(\frac{6B_{1}^{2}a^{8}+15B_{1}B_{2}a^{6}+10a^{4}(2B_{1}B_{3}+B_{2}^{2})+30B_{2}B_{3}a^{2}+30B_{3}^{2}}{30B_{3}^{2}}\right)\;$$

The effective area is necessary to maintain the continuity between the volumetric flow rate of the diaphragm and lumped model. It is used to relate the mechanical domain with the acoustic domain. The volumetric flow (*Q*) is given by:

(A11) $$Q=\int_{0}^{a}v\left(\frac{dP}{dt},r\right)dA=\int_{0}^{a}v\left(\frac{dP}{dt},r\right)2\pi rdr\;$$

The effective area (*A_eff_*) of the lumped element is:

(A12) $$A_{eff}=\frac{Q}{v_{0}}\;$$

The volumetric flow according to Equation (A11) is obtained as:

(A13) $$Q=\left(\frac{\pi a^{4}\left(192C_{s}D\pi (1+v)+a^{2}(v+6\eta +1)\right)}{192D(1+v)}\right)\frac{dP}{dt}\;$$

The effective area *A_eff_* is equal to area of the diaphragm multiplied by a factor called relative area (*A_r_*), which depends on the geometrical dimensions and material properties:

(A14) $$A_{eff}=\pi a^{2}A_{r}\;\;$$

(A15) $$A_{r}=\frac{a^{2}(v+6\eta +1)+192C_{s}D\pi (1+v)}{3a^{2}(v+4\eta +1)+192C_{s}D\pi (1+v)}\;$$

The factor *A_r_* takes into account the deflection of the diaphragm, a rigid plated supported by springs would have a relative area of unity.

With the previous expressions, we can obtain the lumped parameters for a fixed supported diaphragm (*η* = 0, *C_s_* = 0) and a simply supported diaphragm (*η* = 1, *C_s_* = 0), as shown in Table A1.

**Table A1:** Lumped parameters for fixed and simply supported diaphragms.

	Fixed Diaphragm	Simply Supported Diaphragm
Lumped stiffness (*K_m_*)	$\frac{64\pi D}{3a^{2}}$	$\frac{64\pi D\left(1+\nu \right)\left(7+\nu \right)}{3a^{2}{\left(\nu +5\right)}^{2}}$
Lumped mass (*M_m_*)	$\frac{\pi ha^{2}\rho }{5}$	$\frac{\pi ha^{2}\rho \left(3\nu^{2}+36\nu +113\right)}{15{\left(\nu +5\right)}^{2}}$
Relative area (*A_r_*)	$\frac{1}{3}$	$\frac{7+\nu }{3\left(5+\nu \right)}$

## Appendix B

Homentcovschi and Miles [21] presented an analytical model for the viscous damping in perforated planar microstructures. They determined the optimum number of holes for a given value of *ARH*, which ensures equilibrium between squeeze film damping and holes resistance.

The optimum number (*N*) of holes of the backplate is calculated as [21]:

(A16) $$N=a^{2}A_{RH}\sqrt{\frac{3}{2hg^{3}}\left(\frac{A_{RH}}{2}-\frac{A_{RH}^{2}}{8}-\frac{1}{4}\ln\left(A_{RH}\right)-\frac{3}{8}\right)}\;$$

with

(A17) $$A_{RH}=\frac{r_{1}^{2}}{r_{2}^{2}}\;$$

(A18) $$Nr_{2}^{2}=a^{2}\;$$

and the minimum values of the total damping coefficient as [21]:

(A19) $$b_{m}=\pi a^{2}\frac{8\mu \sqrt{6}}{A_{RH}}\sqrt{\frac{h}{g^{3}}\left(\frac{A_{RH}}{2}-\frac{A_{RH}^{2}}{8}-\frac{1}{4}\ln\left(A_{RH}\right)-\frac{3}{8}\right)}\;$$
